# Engineering a norcoclaurine synthase for one-step synthesis of (*S*)-1-aryl-tetrahydroisoquinolines

**DOI:** 10.1186/s40643-023-00637-4

**Published:** 2023-03-01

**Authors:** Man Zhang, Zheng-Yu Huang, Ying Su, Fei-Fei Chen, Qi Chen, Jian-He Xu, Gao-Wei Zheng

**Affiliations:** State Key Laboratory of Bioreactor Engineering, Shanghai Collaborative Innovation Centre for Biomanufacturing, College of Biotechnology, East China University of Science and Technology, Shanghai, 200237 People’s Republic of China

**Keywords:** Biocatalysis, Norcoclaurine synthase, Protein engineering, Pictet–Spengler reaction, Tetrahydroisoquinoline alkaloids

## Abstract

Tetrahydroisoquinoline alkaloids (THIQAs) are ubiquitous compounds with important pharmaceutical and biological activity. Their key *N*-heterocyclic structural motifs are synthesised via Pictet–Spengler (P–S) reaction by norcoclaurine synthases (NCS) in plants. The synthesis of 1-aryl-tetrahydroisoquinoline alkaloids has attracted increasing attention due to their antitumor and antivirus activities. Herein, the L68T/M97V mutant of NCS from *Thalictrum flavum* with improved activity was developed by semi-rational design. This mutant not only showed higher catalytic performance (> 96% conversion) toward benzaldehyde and dopamine over the wild-type enzyme, but also catalysed the P–S reaction of the bulky substrate 4-biphenylaldehyde and dopamine with high conversion (> 99%) for the effective synthesis of 1-aryl-THIQA. In terms of stereoselectivity, all products synthesised by the L68T/M97V mutant showed high optical purity (92–99% enantiomeric excess).

**Graphical Abstract:**

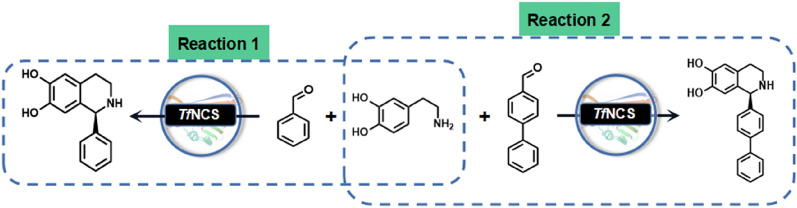

**Supplementary Information:**

The online version contains supplementary material available at 10.1186/s40643-023-00637-4.

## Introduction

**Fig. 1:**
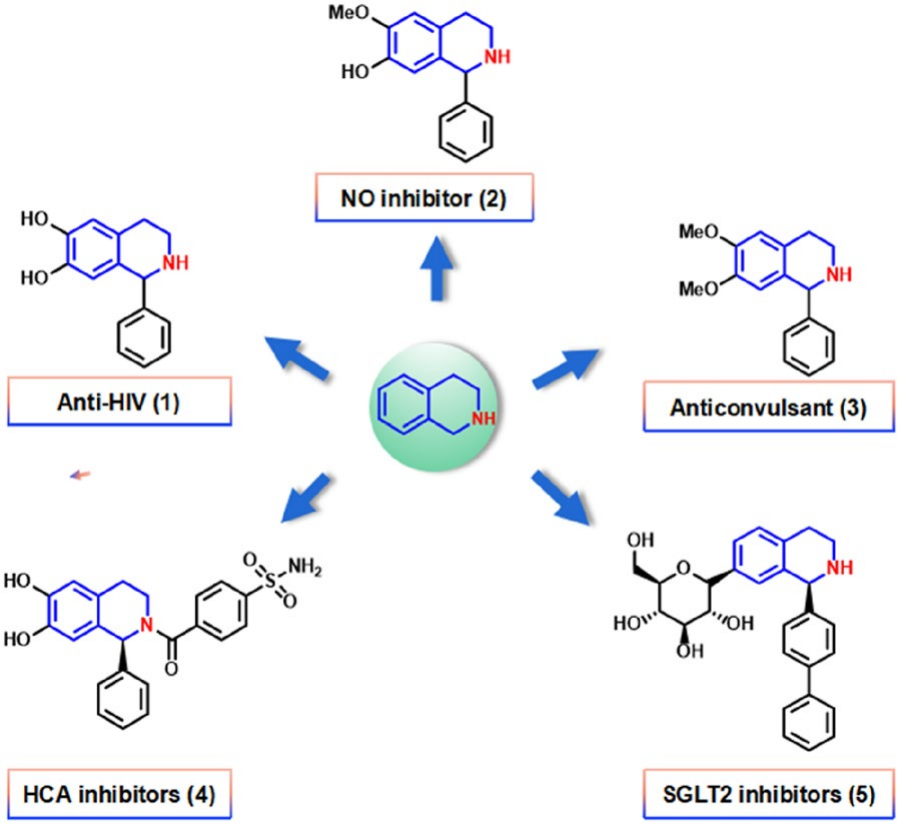
Some representative tetrahydroisoquinoline alkaloids (THIQAs) with pharmacological activities

Tetrahydroisoquinoline alkaloids (THIQAs) are diverse natural compounds with important pharmacological activities, including anaesthesia (Lechner et al. 2018) and antibacterial effects (Samanani et al. 2004). A variety of 1-aryl-THIQAs have been clinically used to treat diseases. For example, 1-phenyl-1,2,3,4-tetrahydroisoquinoline-6,7-diol **1** has anti-HIV activity, and its structural analogue 6-methoxy-1-phenyl-1,2,3,4-tetrahydroisoquinolin-7-ol **2** inhibits the synthesis of nitric oxide to treat some diseases of aging people such as Alzheimer’s disease (Zhu et al. 2017); 6, 7-Dimethoxy-1-phenyl-1,2,3,4-tetrahydroisoquinoline **3** can be used as a precursor to synthesise anticonvulsants (Cheng et al. 2008); 4-(6,7-Dihydroxy-1-phenyl-1,2,3,4-tetrahydroisoquinoline-2-carbonyl)benzenesulfonamide **4** can be used to inhibit some different types of carbonic anhydrases to treat diseases including oedema, glaucoma, cancer, epilepsy and osteoporosis (Bruno et al. 2017); Compound 5 can be used as a sodium-glucose co-transporter 2 (SGLT2) inhibitor (Fig. 1). Chemical synthesis of the main structural motifs of compound 5 requires multi-step reactions and a variety of reagents, resulting in low catalytic efficiency (Pan et al. 2016).

The *N*-heterocyclic structural motif, an important part of all THIQAs, is normally biosynthesised by the Pictet–Spengler (P–S) reaction in plants (Ruff et al. 2012). A number of enzymes catalysing the P–S reaction have been identified, of which norcoclaurine synthase (NCS) is among the most well-studied (Wang et al. 2018; Yamazaki et al. 2003; Yang et al. 2020). NCS catalyses the conversion of dopamine and 4-hydroxyphenylacetaldehyde (4-HPAA) to (*S*)-norcoclaurine, the fundamental compound in biosynthesis of benzylisoquinoline alkaloids (BIAs), and a precursor for the synthesis of more than 2500 BIAs (Dickey et al. 2021; Lee and Facchini 2010; Li et al. 2022; Minami et al. 2007; Tang et al. 2020). Among characterised NCSs, *Tf*NCS isolated from *Thalictrum flavum* is one of the most widely-studied enzymes (Bonamore et al. 2010; Luk et al. 2007; Pasquo et al. 2008; Roddan et al. 2019; Samanani and Facchini 2002). *Tf*NCS shows broad carbonyl substrate scope, extending from aldehyde to ketone substrates (Lichman et al. 2017b; Roddan et al. 2020; Zhao et al. 2021).

Roddan et al. (2020) synthesised similar (*S*)-1-aryl-tetrahydroisoquinolines and analogues via the condensation of dopamine and benzaldehyde catalysed by *Tf*NCS. They also engineered the M97V mutant with higher catalytic activity toward benzaldehyde and its analogues than the wild-type (WT) enzyme. Although the activity of NCS has been improved, it is still inadequate for large-scale applications.

**Fig. 2:**
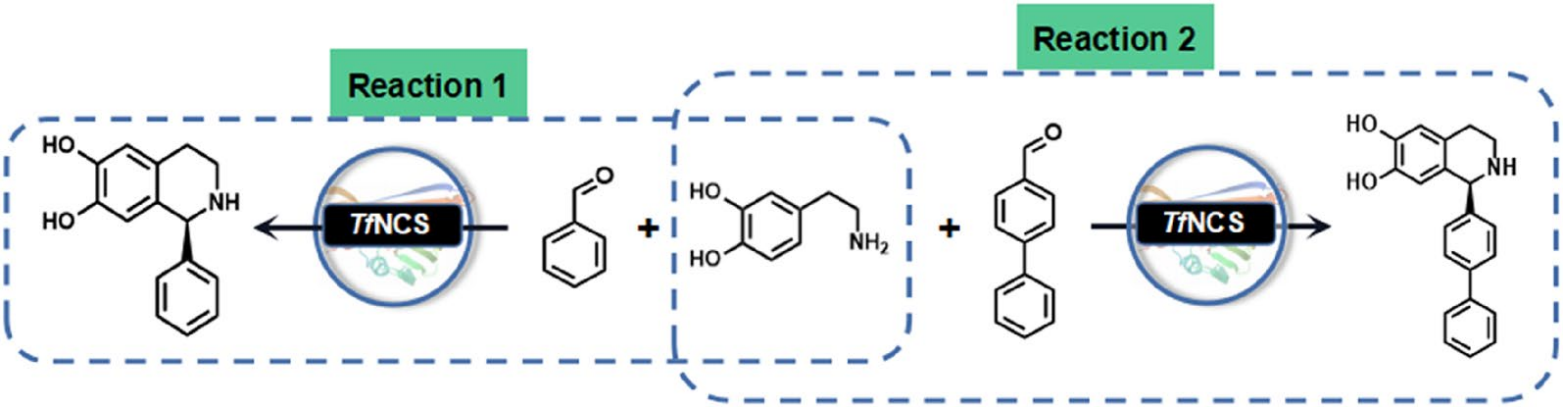
One-step synthesis of 1-aryl-tetrahydroisoquinolines using norcoclaurine synthase (NCS)

Herein, we employed a semi-rational design strategy to improve the activity of *Tf*NCS toward benzaldehyde (Fig. 2), provide a better biocatalyst for the synthesis of 1-aryl-tetrahydroisoquinoline alkaloids. In addition, broadening the substrate specificity of the *Tf*NCS variant toward the bulky 4-biphenylaldehyde was explored, demonstrating the potential for the synthesis of bulky (*S*)-1-aryl- pharmaceutically interesting tetrahydroisoquinoline compounds (Fig. 2).

## Materials and methods

### Transformation and protein expression of TfNCS and its mutants

The codon-optimised gene encoding *Tf*NCS (lacking 19 amino acids from the *N*-terminus) was integrated into the expression vector pET-28a(+) (GenScript, Nanjing, China) and introduced into *Escherichia coli* BL21 (DE3) cells (Novagen, Germany) for protein expression. The mutants mentioned above were obtained using site-directed mutagenesis of the WT gene. For protein expression, a single colony was cultured in Luria–Bertani (LB) medium at 37 °C for 12 h, and then 1 mL fresh culture was transferred into 100 mL Terrific-Broth (TB) medium. A 100 mL volume of TB medium contained with 12 g L^−1^ peptone, 24 g L^−1^ yeast extract, 5 g L^−1^ glycerol, 2.31 g L^−1^ KH_2_PO_4_ and 12.54 g L^−1^ K_2_HPO_4_. The working concentration of kanamycin in TB was 50 µg mL^−1^, and cultures were grown at 37 °C. When the OD_600_ of TB medium reached 0.6–0.8, 0.2 mM isopropyl β-d-thiogalactoside (IPTG) was added and culturing was continued at 16 °C for 20–24 h, cells were collected by centrifugation for subsequent experiments.

### Chemicals

Benzaldehyde, 4-bipenzaldehyde and dopamine were purchased from Aladdin (Shanghai, China), and were of reagent grade or better. PrimeSTAR^HS^ used in site-directed mutagenesis was purchased from TaKaRa Bio. (Dalian, China). Primers required for site-directed mutagenesis were synthesised by Tsingke Bio. (Shanghai, China).

### Protein purification of TfNCS and its mutants

Wet cells were resuspended in buffer A (100 mM NaCl, 100 mM HEPES, 20 mM imidazole, pH 7.5) (Lichman et al. 2017b), and cell suspensions were lysed in an ice bath using an JY92-II ultrasonic oscillator (Scientz Biotech Co.) with 4 s pulses and 6 s pauses at 20% amplitude for 15 min. Cell lysates were centrifuged (12,000 rpm, 1 h, 4 °C) to obtain clarified supernatants. The filtered supernatant was passed through a 5 mL Ni–NTA column (GE Healthcare, America) previously equilibrated with deionised water and buffer A (1.5-fold column volumes). Bound *Tf*NCS protein and its mutants were eluted with 70% buffer B (100 mM NaCl, 100 mM HEPES, 500 mM imidazole, pH 7.5) and collected in 10 kDa ultrafiltration tubes. The eluent buffer containing pure enzyme was exchanged for assay buffer (50 mM NaCl, 20 mM Tris, pH 7.5). The protein concentration of purified enzyme was determined by a Nanodrop 2000c spectrophotometer (Thermo Scientific, America) at 280 nm, and the extinction coefficient was calculated using the ExPaSy ProtParam Tool (Gasteiger et al. 2005).

### Enzymatic activity assay

Activity assays of *Tf*NCS and its mutants were performed in 2 mL Eppendorf tubes on MHR23 mini-shaker (HLC BioTech, Germany). For reaction condition 1 (crude enzyme, as shown in Additional file 1: Fig. S1), the reaction mixture (500 μL) was composed of 10 mM dopamine, 1 mM benzaldehyde, 20% (v/v) clarified lysate of *Tf*NCS and its mutants, 20% (v/v) dimethylsulphoxide (DMSO), and 5 mM ascorbic acid in HEPES buffer (pH 7.5, 100 mM). Reactions were performed for 24 h at 30 °C with shaking at 1000 rpm (Lichman et al. 2017b). For reaction condition 2 (purified enzyme, as shown in Figs. 5 and 6), the reaction mixture (500 μL) was composed of 10 mM amine, 1 mM aldehyde, 0.5 mg purified enzyme (benzaldehyde) or 4 mg purified enzyme (4-bipenzaldehyde), 20% (v/v) DMSO, and 5 mM ascorbic acid in HEPES buffer (pH 7.5, 100 mM). Reactions were performed at 40 °C and 1000 rpm. For reaction condition 3 (purified enzyme, as shown in Table 1 and Fig. 7), the reaction mixture (500 μL) was composed of 2 eq. amine, 1 eq. aldehyde, 1 mg purified enzyme and benzaldehyde or 4 mg purified enzyme and 4-bipenzaldehyde, 20% (v/v) DMSO, and 5 mM ascorbic acid in HEPES buffer (pH 7.5, 100 mM). Reactions were performed at 40 °C and 1000 rpm (Roddan et al. 2020).

**Table 1:** Asymmetric synthesis of THIQAs at different temperatures with benzaldehyde and dopamine as substrates

Entry	°C	Conversion^[a]^ (%)			*ee*^[b]^ (%)	
		Blank	WT	L68T/M97V	WT	L68T/M97V
1	25	6	35	84	85	95
2	30	10	53	96	97	98
3	40	11	56	97	92	92

The reaction mixture was composed of 50 mM amine, 25 mM aldehyde, 1 mg purified enzyme loaded with benzaldehyde, 20% (v/v) DMSO, 5 mM ascorbic acid and HEPES buffer (100 mM, pH 7.5)

All reactions were performed at 40 °C for 3 h, in duplicate ^[a][b]^, and analysed by HPLC

### High performance liquid chromatography (HPLC) analysis

Using achiral HPLC, conversions catalysed by *Tf*NCS and its mutans were determined at 280 nm (30 °C). The LC-2010A HPLC instrument (Shimadzu, Japan) was equipped with a Hypersil ODS2 C-18 column (250 mm × 4.6 mm, 5 μm particle size; Elite, China) and a UV detector. Acetonitrile (MeCN) with 1% (v/v) trifluoroacetic acid (TFA) and water (H_2_O) with 1% (v/v) TFA were used as mobile phase. The gradient applied for separation was 10% MeCN for 1 min, a linear gradient to 90% MeCN over 8 min, 90% MeCN for 7 min, a linear gradient to 10% MeCN over 8 min, and holding for 3 min. The flow rate was 0.6 mL min^−1^.

Using chiral HPLC analysis, enantiomeric excess (*ee*) values of *Tf*NCS and its mutans were determined at 280 nm (40 °C). The LC-2010A HPLC instrument (Shimadzu) was equipped with a Hypersil ODS2 AD-H column (250 mm × 4.6 mm, 5 μm particle size; Elite) and a UV detector. 1 M NaHCO_3_ (one-fold reaction volume) and ethyl acetate (twofold reaction volume) were successively added to the enzymatic reaction for extraction. Finally, the upper ethyl acetate layer was collected, mixed with anhydrous Na_2_SO_4_, and dried for more than 12 h. The mobile phase was composed of ethanol with 1‰ (v/v) diethylamine and *n*-hexane with 1‰ (v/v) diethylamine at a flow rate of 0.6 mL min^−1^. For benzaldehyde, the dry samples were eluted with a 60:40 (v/v) mobile phase. For, 4-biphenylcarboxaldehyde, the dry samples were eluted with a 4:6 ratio of mobile phase.

### Bioinformatics methods

To probe the volume of the substrate-binding pocket, the structure of the L68T/M97V mutant was modelled by AlphaFold2 (Humphreys et al. 2021). The resulting structure of the L68T/M97V mutant was used to dock the imine intermediate formed by benzaldehyde and dopamine with AutoDock (Forli et al. 2016). Structures were assessed using PyMol (Molecular Graphics System, Version 2.0, Schrçdinger, LLC, 2017). For calculation of the volume of the substrate-binding pocket we used POCASA 1.1 (Yu et al. 2010), an online tool for predicting protein pockets. We imported the PDB file of WT *Tf*NCS without the ligand and the PDB file of the L68T/M97V mutant generated by modelling. We set the parameters Grid Size, Probe radius, Single Point Flag, Protein Depth Flag, and Chain ID to 0.5 Å, 3 Å, 9, 10 and null, respectively. The number of pockets was set to five and ‘Get Pockets and Cavities’ was selected. We downloaded the resulting file and used Pymol to determine the specific pocket position. We then compared the interaction energies using Ligplus software (Nan et al. 2021). We mainly investigated changes in hydrogen bonding and hydrophobic interactions around the pocket when the imine intermediate was bound.

### Determination of kinetic parameters

Kinetic parameters of WT *Tf*NCS and the L68T/M97V mutant with benzaldehyde and 4-biphenylaldehyde were determined by measuring the conversion at different substrate concentrations (0.5–50 mM). Activity was assayed by HPLC and samples were tested in duplicate. All data were analysed by Graphpad Prism 9.0 using the Michaelis–Menten model.

## Results and discussion

### Engineering *Tf*NCS to improve catalytic activity

**Fig. 3:**
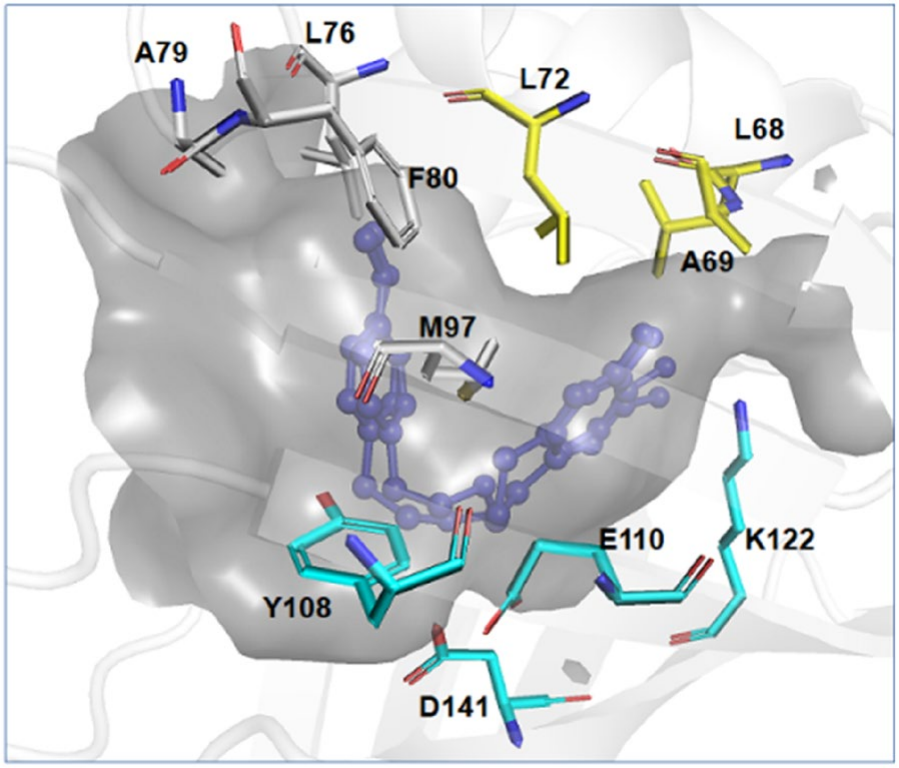
The substrate-binding pocket of *Tf*NCS (PDB ID: 5NON). Catalytic residues and the product mimic are coloured cyan and blue, respectively. Residues around the aldehyde substrate and the hydroxyl group of the amine substrate are coloured white and yellow, respectively

Based on the structure of *Tf*NCS complexed with the product mimic (PDB ID: 5NON) reported in the literature (Lichman et al. 2017a), we analysed the substrate-binding pocket of *Tf*NCS based on the two substrates. Residues L76, A79, F80 and M97 around the aldehyde substrate and L68, A69 and L72 around the hydroxyl group of the amine substrate may influence binding between enzyme and substrates, hence they were considered hotspots for mutagenesis (Fig. 3).

At the outset, a site-directed saturation mutagenesis strategy was employed on residues around the amine substrate (L68, A69, L72), and reported hotspot mutations (L76V, M97V, A79F, A79I and F80L) (Lichman et al. 2017b) around the aldehyde substrate were tested. The activity of the variants generated from the first round of mutagenesis was screened using benzaldehyde and dopamine as model substrates. Mutants L68T, L68S, A69R, L72P and M97V displayed good activity toward benzaldehyde, and the activity of mutant L68T was significantly improved compared with that of the WT enzyme (Additional file 1: Fig. S1). This result encouraged us to choose the mutant L68T as the template for subsequent mutagenesis.

**Fig. 4:**
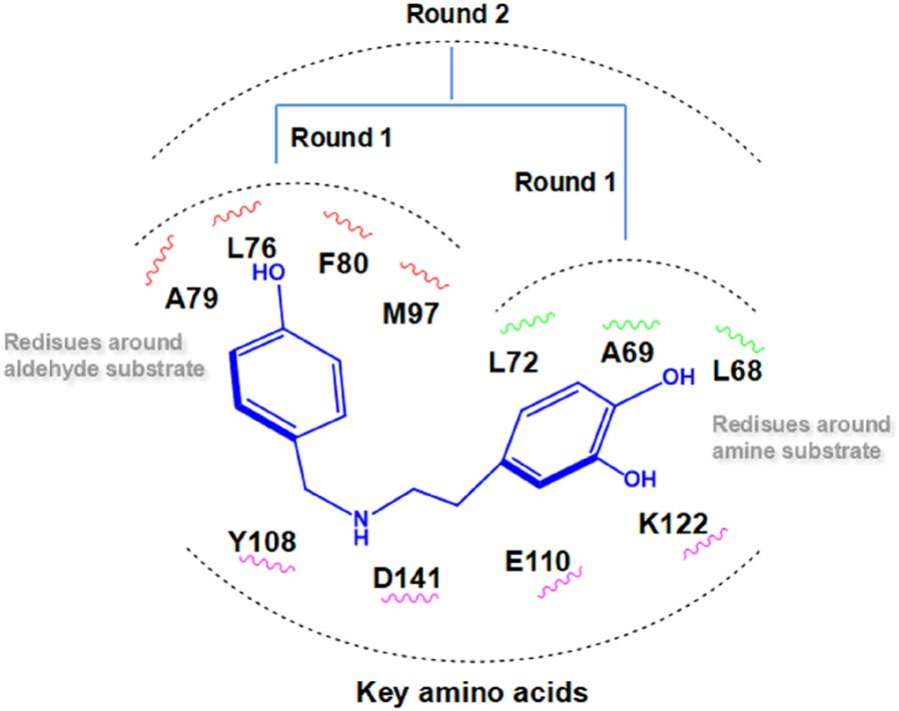
Schematic diagram of the mutagenesis strategy

**Fig. 5:**
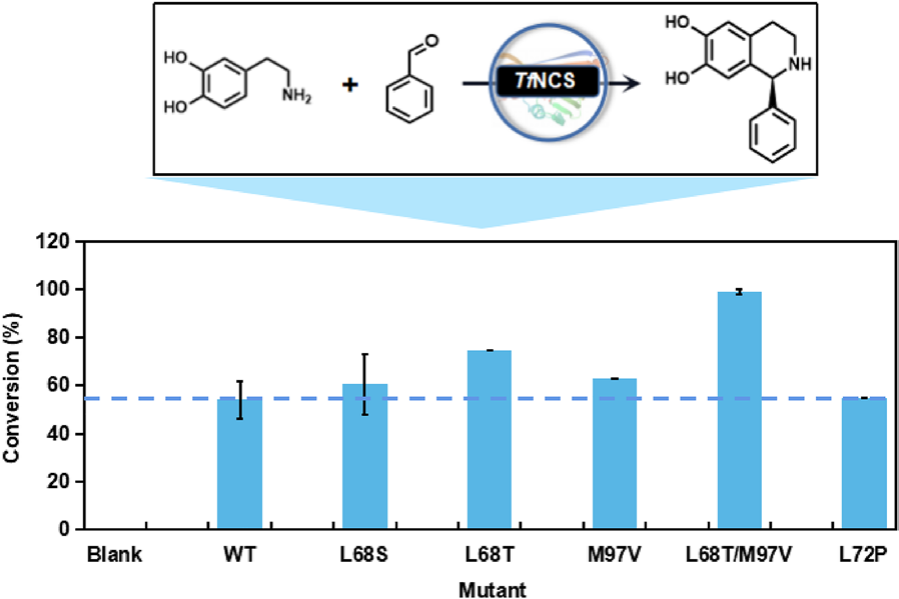
Biotransformation of benzaldehyde and dopamine catalysed by *Tf*NCS and its mutants. The reaction mixture was composed of 10 mM amine, 1 mM aldehyde, 20% (v/v) DMSO, 5 mM ascorbic acid, HEPES buffer (100 mM, pH 7.5) and 0.5 mg mL^−1^ purified enzyme. Reactions were performed at 30 °C for 3 h

Considering the possible synergistic effects between residues surrounding the pocket, we subsequently constructed a combinatorial mutant library using L68T from the first round of mutagenesis as the parent, combined with other hotspot residues around the pocket (L68S, A69R, L72P, L76V, M97V, A79F, A79I and F80L) (Lichman et al. 2017b) (Fig. 4). Mutant L68T/M97V showed the best catalytic activity, significantly improved compared with WT enzyme for reaction with benzaldehyde and dopamine (Additional file 1: Fig. S1). The positive mutants L68T, L68S, L72P, M97V and L68T/M97V obtained from rounds 1 and 2 were purified and used to further confirm their ability for biotransformation of benzaldehyde and dopamine. As shown in Fig. 5, mutant L68T/M97V exhibited the highest activity, achieving 99% conversion, an almost two-fold improvement over the WT enzyme. This was generally consistent with the screening results for cell-free extracts.

This reaction had a high background with benzaldehyde and its analogues as aldehyde substrates (Roddan et al. 2020). Thus, the reaction conditions were optimised to achieve higher conversion and *ee*. The optimal reaction temperature was first investigated by performing reactions with dopamine and benzaldehyde at different temperatures (Table 1). The background reaction was increased with higher reaction temperature (30 °C and 40 °C), which was not conducive to obtaining products with high stereospecificity. Thus, the reaction temperature in subsequent experiments was no higher than 40 °C.

In addition, mutant L68T/M97V showed higher conversion and product enantiopurity after 3 h compared with WT enzyme at different temperatures; Conversion reached 96%, nearly two-fold higher than WT enzyme (53%). This mutant also achieved high product stereoselectivity (92–98% *ee*). When loaded with benzaldehyde as the aldehyde substrate the yield reached 25 mM, a 2.5-fold improvement compared with the previously reported value (Roddan et al. 2020). This highlighted the feasibility of our mutagenesis strategy, with the catalytic activity of mutant L68T/M97V toward benzaldehyde reach the highest compared with the reported to date.

### Asymmetric synthesis of 1-aryl-THIQAs

**Fig. 6:**
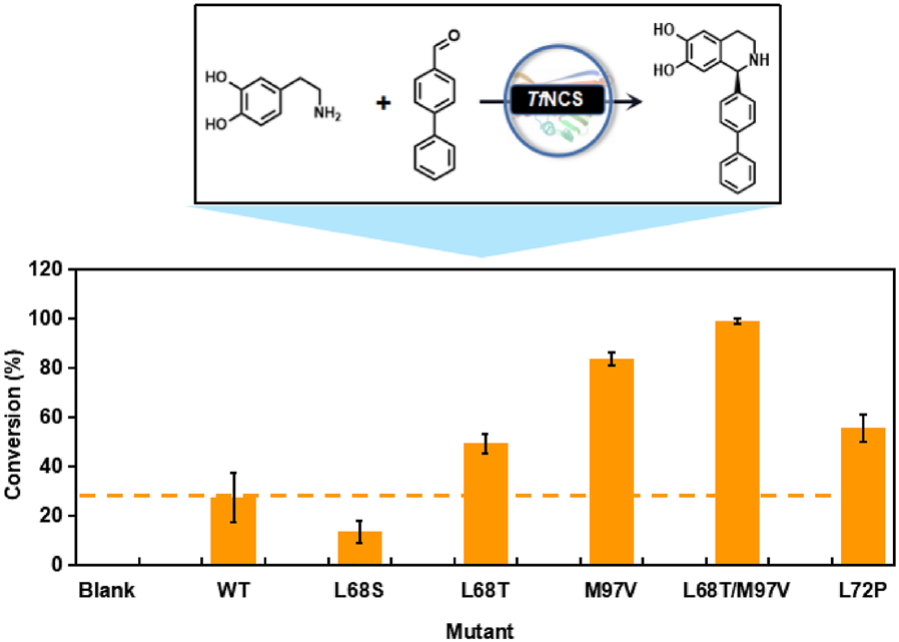
Biotransformation of 4-bipenzaldehyde and dopamine catalysed by *Tf*NCS and its mutants. The reaction mixture was composed of 10 mM amine, 1 mM aldehyde, 20% (v/v) DMSO, 5 mM ascorbic acid, HEPES buffer (100 mM, pH 7.5) and 4 mg mL^−1^ purified enzyme. Reactions were performed at 30 °C for 24 h

To explore the viability of *Tf*NCS for the synthesis of bulky unnatural 1-aryl-THIQAs, we attempted the biotransformation of dopamine and the bulky 4-bipenzaldehyde, a challenging substrate for NCS. We initially explored the reactivity of these positive mutants with 4-bipenzaldehyde and dopamine. As shown in Fig. 6, almost all mutants tested (except for L68S) displayed improved reactivity over WT. Among them, the L68T/M97V mutant achieved the highest conversion, up to 99% within 24 h, indicating that it may be a promising biocatalyst for biotransformation of bulky aldehydes.

**Fig. 7:**
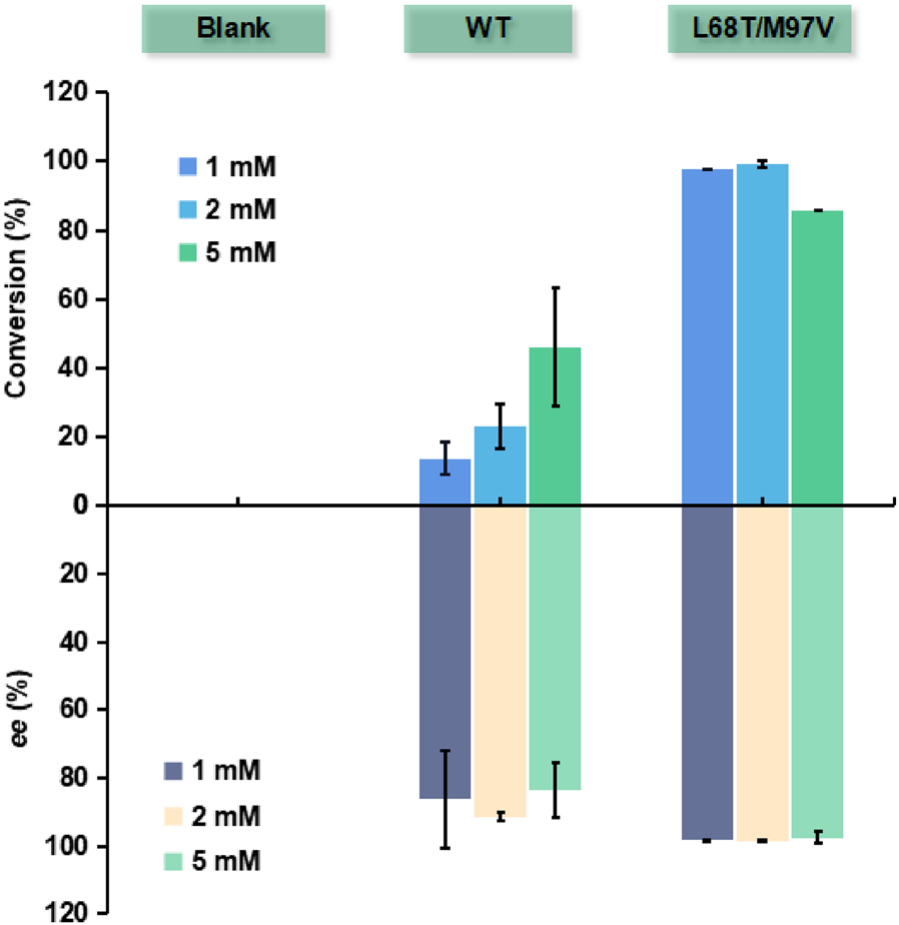
Asymmetric synthesis of bulky THIQAs using 4-bipenzaldehyde and dopamine as substrates. The reaction mixture was composed of 2 eq. amine, 1 eq. aldehyde, 20% (v/v) DMSO, 5 mM ascorbic acid, HEPES buffer (100 mM, pH 7.5) and 4 mg mL^−1^ purified enzyme. Reactions were performed at 40 °C for 24 h

Subsequently, we also explored the tolerance of mutant L68T/M97V at different substrate loadings (Fig. 7). Mutant L68T/M97V exhibited up to 99% conversion at low substrate loading, compared with < 20% for WT enzyme. In addition, mutant L68T/M97V displayed improved *ee* over WT enzyme at various substrate loadings, up to 98%. For the bulky 4-biphenylaldehyde substrate, this is the first *Tf*NCS enzyme known to catalyse its conversion. The catalytic activity and stereoselectivity of mutant L68T/M97V were significantly better than for WT enzyme. The acquisition of new mutant L68T/M97V provided a new addition of NCS enzymes for the biocatalytic synthesis of useful tetrahydroisoquinoline alkaloids.

### The molecular mechanism underpinning the increased activity

In order to analyse the mechanism for the improved activity of mutant L68T/M97V, kinetic parameters WT *Tf*NCS and the L68T/M97V mutant with benzaldehyde and 4-bipenzaldehyde were measured using HPLC. As shown in Table 2, the *K*_m_ value of mutant L68T/M97V was decreased slightly from 22.26 to 20.11 mM for benzaldehyde, and from 15.04 to 14.61 mM for 4-bipenzaldehyde, compared with WT enzyme. The *k*_cat_ of mutant L68T/M97V was 0.439 s^−1^ for benzaldehyde and 0.002 s^−1^ for 4-bipenzaldehyde, two-fold higher than for WT (0.237 s^−1^ for benzaldehyde and 0.001 s^−1^ for 4-bipenzaldehyde). This resulted in a two-fold increase in catalytic efficiency (*k*_cat_/*K*_m_) compared with WT enzyme. These results preliminarily explain the reason for improved reactivity of mutant L68T/M97V.

**Table 2:** Kinetic parameters of *Tf*NCS and mutant L68T/M97V toward benzaldehyde and 4-biphenylaldehyde

Enzyme	Aldehyde	*k*_cat_ (s^−1^)	*K*_m_ (mM)	*k*_cat_/*K*_m_ (s^–1^ μM^−1^)
WT	Benzaldehyde	0.237 ± 0.028	22.3 ± 6.7	10.6
L68T/M97V		0.439 ± 0.047	20.1 ± 5.3	21.8
WT	4-Biphenylaldehyde	0.001 ± 0.0001	15.0 ± 4.6	0.06
L68T/M97V		0.002 ± 0.0002	14.6 ± 5.1	0.14

Kinetic parameters were analysed using achiral HPLC

**Fig. 8:**
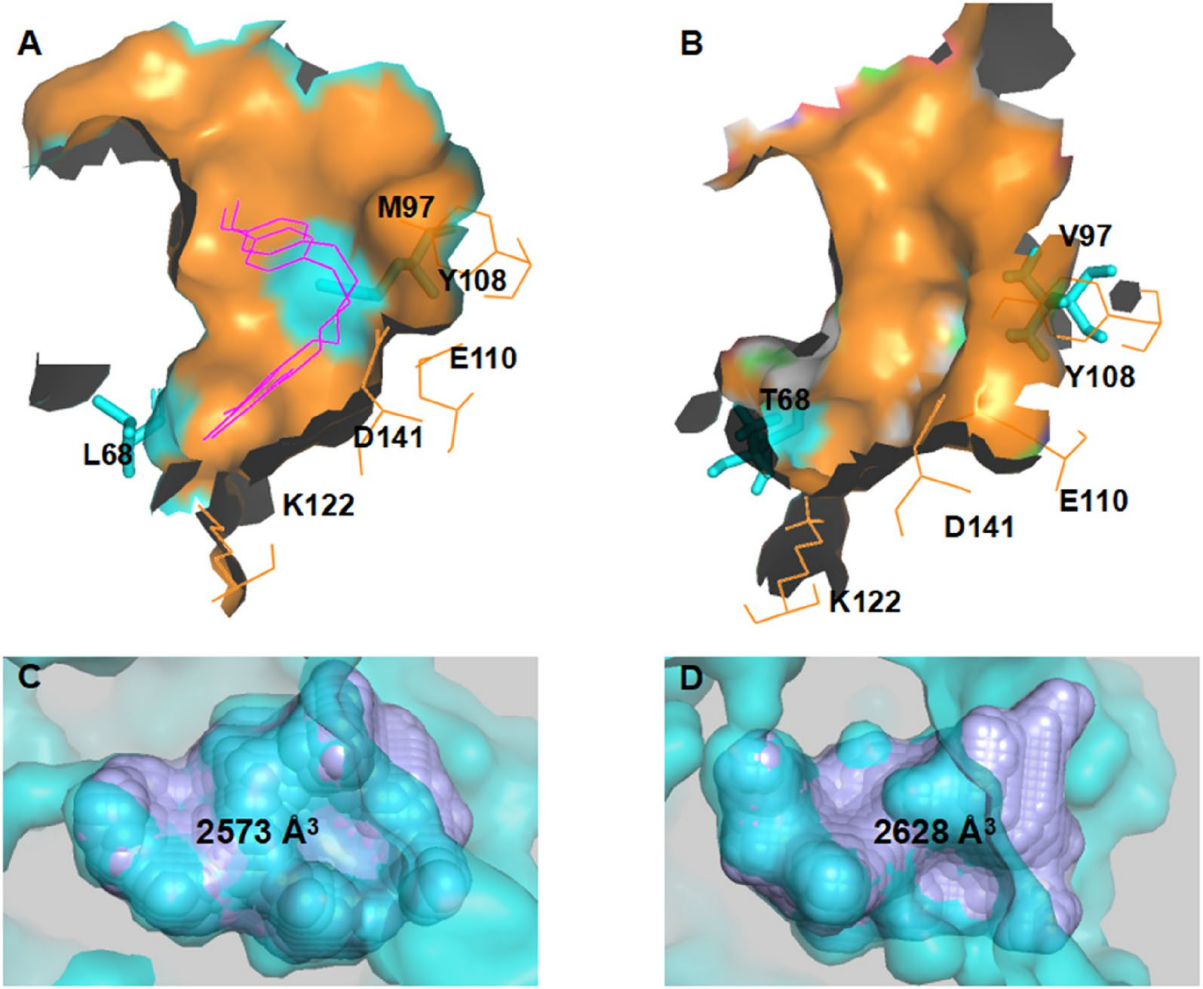
Structural analysis of the substrate-binding pocket of *Tf*NCS and its mutant L68T/M97V using POCASA 1.1. **A** Sectional view of the substrate pocket in *Tf*NCS. **B** Sectional view of the substrate pocket in mutant L68T/M97V modelled by AlphaFold2. **C** Volume of the substrate-binding pocket in the WT enzyme. **D** Volume of the substrate-binding pocket in mutant L68T/M97V

Subsequently, we used bioinformatics tools including POCASA 1.1 (Yu et al. 2010) to further explore the structural mechanism of increased activity. We firstly compared changes in the substrate-binding pocket between WT *Tf*NCS and mutant L68T/M97V. Based on a sectional view of the substrate pocket, replacement of leucine (L) at position 68 with threonine (T) was found to form a new cavity lacking in the original pocket (Fig. 8A, B). Meanwhile, changing the residue at position 97 from methionine (M) to valine (V) also increased the volume of the substrate-binding pocket of *Tf*NCS. This result was verified by calculating the volume of the pockets. After mutation, the volume of the pocket in mutant L68T/M97V was increased by 55 Å^3^ compared with WT enzyme (Fig. 8C, D). The increased volume of the substrate-binding pocket may facilitate binding of the bulky 4-biphenylaldehyde substrate, resulting in the improved catalytic efficiency of mutant L68T/M97V.

**Fig. 9:**
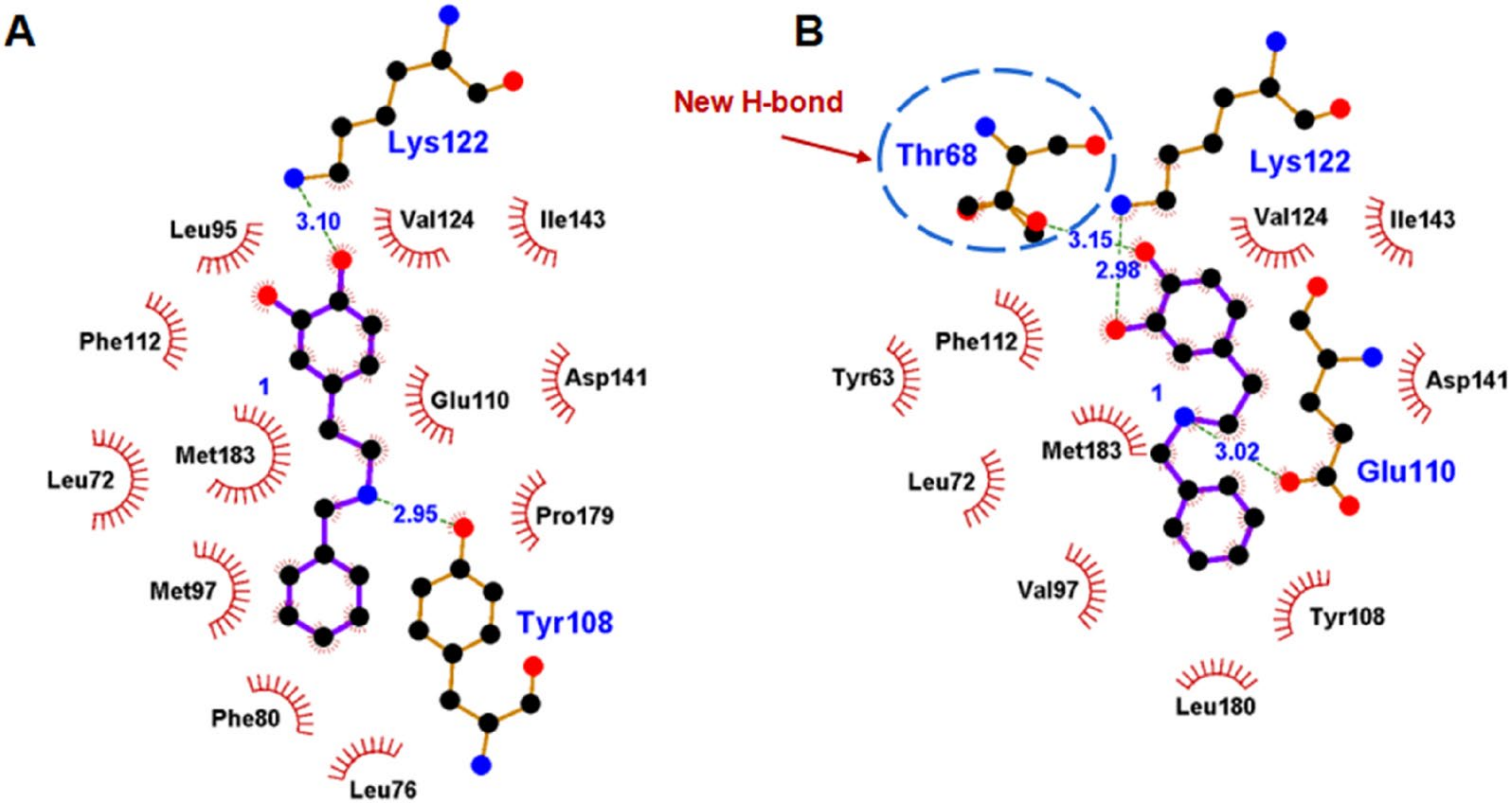
Analysis of interactions around the substrate pocket of WT *Tf*NCS and its mutant L68T/M97V using Ligplus. **A** Hydrogen bonds and hydrophobic interactions between substrates and residues in the binding pocket of WT *Tf*NCS. **B** Hydrogen bonds and hydrophobic interactions between substrates and residues in the binding pocket of mutant L68T/M97V

Finally, changes in hydrogen bonds and hydrophobic interactions around the imine intermediate were also investigated using LigPlus (Nan et al. 2021). Compared with WT *Tf*NCS (Fig. 9A), a new hydrogen bond is formed between T68 and the substrate in mutant L68T/M97V (Fig. 9B), which is beneficial to enhance the binding affinity of the substrate. In addition, the docking energy was decreased from − 7.1 to − 7.9 kcal mol^−1^ following mutation, indicating a more stable docking conformation.

## Discussion

A large number of bioactive molecules containing *N*-heterocycle structural motifs, such as THIQAs, have significant analgesic and anti-cancer effects. In recent years, biosynthesis of THIQAs has attracted the interest of many researchers. The natural *N*-heterocyclic structural motifs are typically synthesised by enzymes that catalyse the P–S reaction. NCS in plants is among the most widely used P–S enzymes. In the present work, we improved the catalytic activity of *Tf*NCS using a semi-rational design strategy to modify *Tf*NCS from *T. flavum*, to improve the turnover of 4-biphenylaldehyde, an bulky aldehyde substrate that has not been successfully utilised. This study lays a foundation for future mining of new drugs and industrial applications for THIQAs.

Researchers have modified NCS in previous work, and this expanded its carbonyl substrate scope. Lichman et al. (2017a, b) achieved the efficient catalysis of aromatic ketones by *Tf*NCS by employing a molecular modification strategy, which promoted the synthesis of helical THIQAs (Lichman et al. 2017b). Roddan et al. (2020) improved the catalytic activity of *Tf*NCS with benzaldehyde and its structural analogues through protein engineering, and synthesised (*S*)-1-aryl-THIQAs possessing medicinal value (Roddan et al. 2020). Zhao et al. (2021) achieved efficient catalysis of *Tf*NCS with methyl-substituted ketones, bicyclic ketones and diketones, which greatly expanded the ketone substrate scope of the enzyme (Zhao et al. 2021). These studies show that molecular modification can improve the substrate specificity of NCS enzymes.

However, protein engineering of NCS has been limited to small, sterically hindered aldehyde substrates, while researches on bulky aldehyde substrates remain scarce. In this study, we initially focused on the residues around the substrate-binding pocket that may affect catalytic activity of NCS based on the types of typical catalytic substrates. Then a site-directed saturation mutagenesis strategy was performed on these residue sites to obtain mutants with improved the transformation efficiency of *Tf*NCS. Meanwhile, we explored the catalytic activity of positive mutants with the bulky aldehyde 4-bipenzaldehyde, and achieved high conversion (> 99%). We hope that researchers will explore the catalytic activity of the optimal L68T/M97V mutant with even bulkier aldehyde substrates in the future.

We also explored the effect of reaction temperature on the background reaction when *Tf*NCS processes highly activated aldehydes. The background reaction was also optimised by altering the pH of the reaction mixture in previous work (Roddan et al. 2020). Finally, the possible reasons for increased activity of mutant L68T/M97V were investigated based on kinetic parameters, changes in the volume of the enzyme pocket, and differences in interactions in the active site, providing useful information for future engineering of NCS.

## Conclusions

Through two rounds of molecular modification, we developed mutant L68T/M97V with improved reactivity and stereoselectivity over WT *Tf*NCS. This mutant displayed improved catalytic activity not only toward benzaldehyde, but also bulky 4-biphenylaldehyde. Thus, it is a promising biocatalyst for the synthesis of 1-aryl-THIQAs.

### Supplementary Information

**Additional file 1: Figure S1.** Relative activity of *Tf*NCS and its mutants towards benzaldehyde and dopamine. Reactions were performed for 24 h using crude enzyme. Analysis of activity by achiral HPLC. **Figure S2.** The SDS-PAGE analysis of target proteins (*Tf*NCS and L68T/M97V). M: Marker. Lane 1, the purified enzyme of *Tf*NCS after being concentrated. Lane 2, collection liquid of *Tf*NCS before being concentrated. Lane 3 and lane 4, cell-free extract and precipitate of *Tf*NCS. Lane 5 and lane 6, cell-free extract and precipitate of mutant L68T/M97V. Lane 7, the purified enzyme of mutant L68T/M97V after being concentrated. **Figure S3.** Achiral HPLC analysis of *Tf*NCS and its mutant toward dopamine and benzaldehyde. **Figure S4.** Achiral HPLC analysis of *Tf*NCS and mutant L68T/M97V toward dopamine and 4-biphenylaldehyde. **Figure S5.** Chiral HPLC analysis of *Tf*NCS and mutant L68T/M97V toward dopamine and benzaldehyde. **Figure S6.** Chiral HPLC analysis of *Tf*NCS and mutant L68T/M97V toward dopamine and 4-biphenylaldehyde. **Figure S7.** Michaelis–Menten-plots towards aldehydes by *Tf*NCS and mutant L68T/M97V. **Figure S8.** ^1^H and ^13^C NMR spectra and data of the catalytic product.

## Data Availability

All data generated or analysed during this study are included in this article and its additional information file.
