# Supplementary material for: Engineering a norcoclaurine synthase for one-step synthesis of (S)-1-aryl-tetrahydroisoquinolines
Source: Bioresour Bioprocess. 2023 Mar 1;10(1):15. doi: 10.1186/s40643-023-00637-4 (PMC10992437; doi:10.1186/s40643-023-00637-4)
Supplement: Supplementary file 1 — Additional file 1: Figure S1. Relative activity of TfNCS and its mutants towards benzaldehyde and dopamine. Reactions were performed for 24 h using crude enzyme. Analysis of activity by achiral HPLC. Figure S2. The SDS-PAGE analysis of target proteins (TfNCS and L68T/M97V). M: Marker. Lane 1, the purified enzyme of TfNCS after being concentrated. Lane 2, collection liquid of TfNCS before being concentrated. Lane 3 and lane 4, cell-free extract and precipitate of TfNCS. Lane 5 and lane 6, cell-free extract and precipitate of mutant L68T/M97V. Lane 7, the purified enzyme of mutant L68T/M97V after being concentrated. Figure S3. Achiral HPLC analysis of TfNCS and its mutant toward dopamine and benzaldehyde. Figure S4. Achiral HPLC analysis of TfNCS and mutant L68T/M97V toward dopamine and 4-biphenylaldehyde. Figure S5. Chiral HPLC analysis of TfNCS and mutant L68T/M97V toward dopamine and benzaldehyde. Figure S6. Chiral HPLC analysis of TfNCS and mutant L68T/M97V toward dopamine and 4-biphenylaldehyde. Figure S7. Michaelis–Menten-plots towards aldehydes by TfNCS and mutant L68T/M97V. Figure S8. 1H and 13C NMR spectra and data of the catalytic product. [file 40643_2023_637_MOESM1_ESM.docx]

**Additional Information**

**Engineering a Norcoclaurine Synthase for One-step Synthesis of (*S*)-1-aryl-Tetrahydroisoquinolines**

**Man Zhang, Zheng-Yu Huang, Ying Su, Fei-Fei Chen, Qi Chen, Jian-He Xu, and Gao-Wei Zheng***

^a^State Key Laboratory of Bioreactor Engineering, Shanghai Collaborative Innovation Centre for Biomanufacturing, College of Biotechnology, East China University of Science and Technology, Shanghai 200237, P. R. China.

^*^ Corresponding author:

Prof. Gao-Wei Zheng, email: gaoweizheng@ecust.edu.cn

**> *Tf*NCS**

CAGAAGCTGATCCTGACCGGTCGTCCGTTCCTGCACCACCAGGGCATCATTAACCAAGTGAGCACCGTTACCAAAGTGATCCACCACGAGCTGGAAGTTGCGGCGAGCGCGGACGATATTTGGACCGTGTACAGCTGGCCGGGTCTGGCGAAGCACCTGCCGGACCTGCTGCCGGGCGCGTTCGAGAAACTGGAAATCATTGGTGACGGTGGCGTTGGCACCATCCTGGATATGACCTTCGTGCCGGGTGAATTTCCGCACGAGTACAAGGAAAAATTTATCCTGGTTGACAACGAACACCGTCTGAAGAAAGTGCAAATGATTGAGGGTGGCTATCTGGACCTGGGTGTTACCTACTATATGGATACCATCCACGTGGTTCCGACCGGCAAGGATAGCTGCGTTATTAAAAGCAGCACCGAGTATCACGTGAAGCCGGAATTTGTTAAAATCGTGGAACCGCTGATTACCACCGGTCCGCTGGCGGCGATGGCGGACGCGATCAGCAAGCTGGTTCTGGAGCACAAGAGCAAAAGCAACAGCGATGAGATTGAAGCGGCGATCATTACCGTGTAA

**
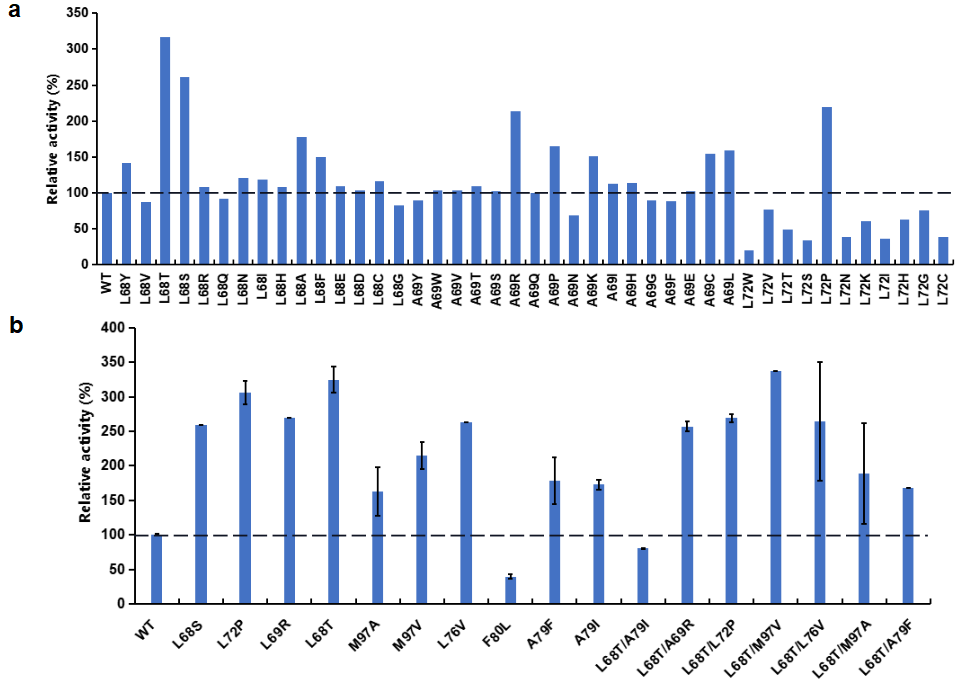
**

**Fig. S1** Relative activity of *Tf*NCS and its mutants towards benzaldehyde and dopamine. Reactions were performed for 24 h using crude enzyme. Analysis of activity by achiral HPLC


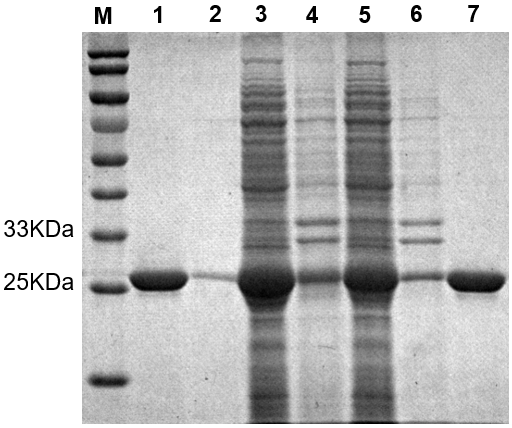


**Fig. S2** The SDS-PAGE analysis of target proteins (*Tf*NCS and L68T/M97V). M: Marker. Lane 1, the purified enzyme of *Tf*NCS after being concentrated. Lane 2, collection liquid of *Tf*NCS before being concentrated. Lane 3 and lane 4, cell-free extract and precipitate of *Tf*NCS. Lane 5 and lane 6, cell-free extract and precipitate of mutant L68T/M97V. Lane 7, the purified enzyme of mutant L68T/M97V after being concentrated.


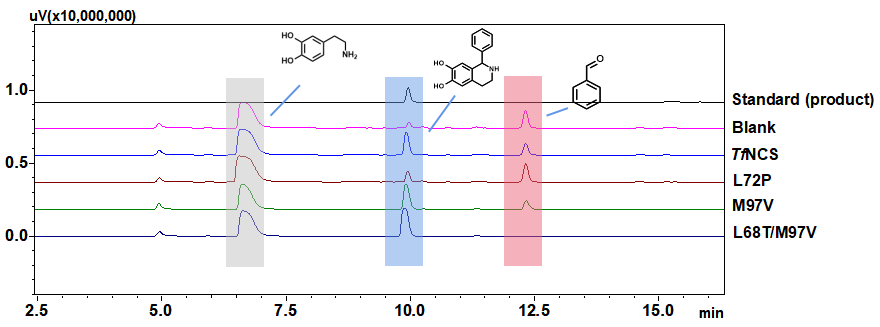


**Figure S3** Achiral HPLC analysis of *Tf*NCS and its mutant toward dopamine and benzaldehyde.


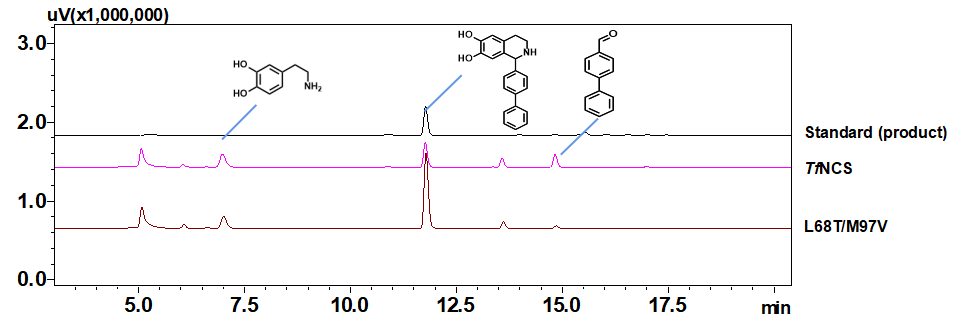


**Figure S4** Achiral HPLC analysis of *Tf*NCS and mutant L68T/M97V toward dopamine and 4-biphenylaldehyde.


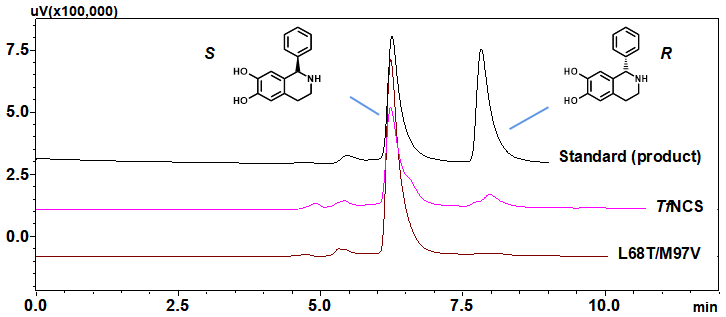


**Figure S5** Chiral HPLC analysis of *Tf*NCS and mutant L68T/M97V toward dopamine and benzaldehyde.


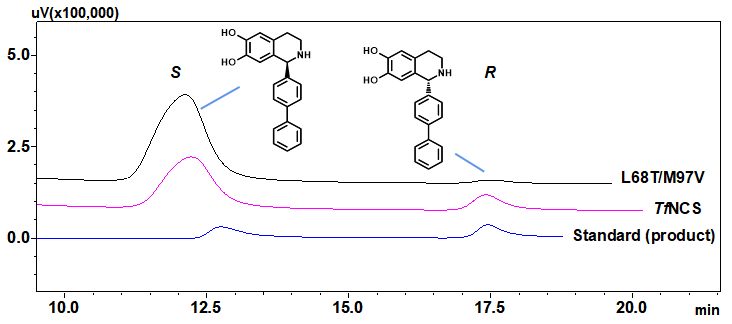


**Figure S6** Chiral HPLC analysis of *Tf*NCS and mutant L68T/M97V toward dopamine and 4-biphenylaldehyde.

**
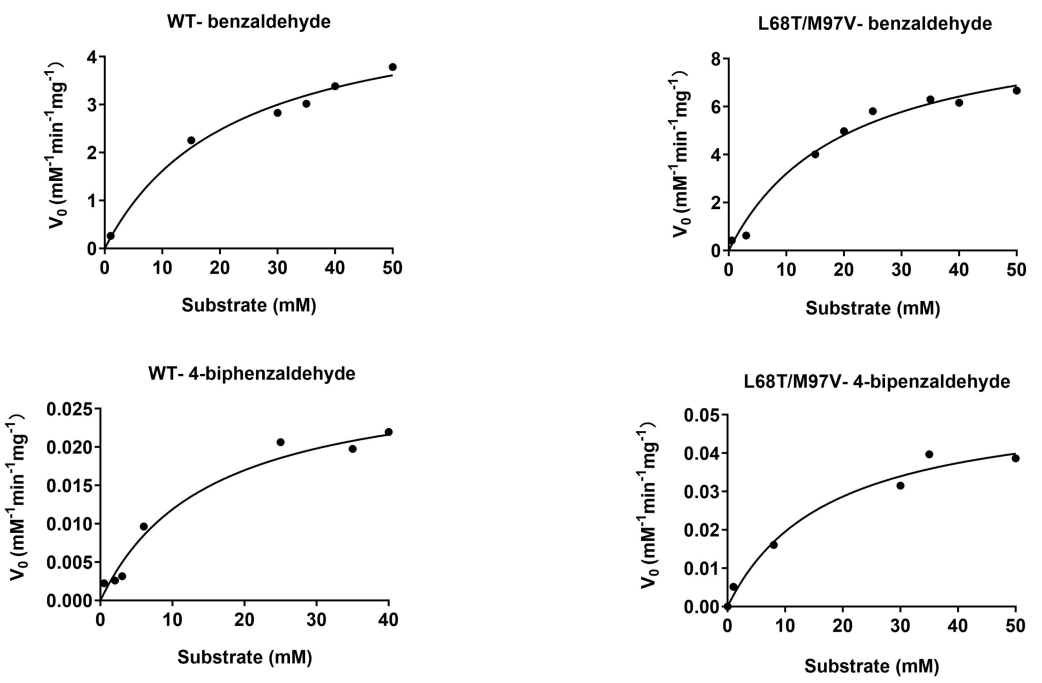
**

**Figure S7** Michaelis-Menten-plots towards aldehydes by *Tf*NCS and mutant L68T/M97V.


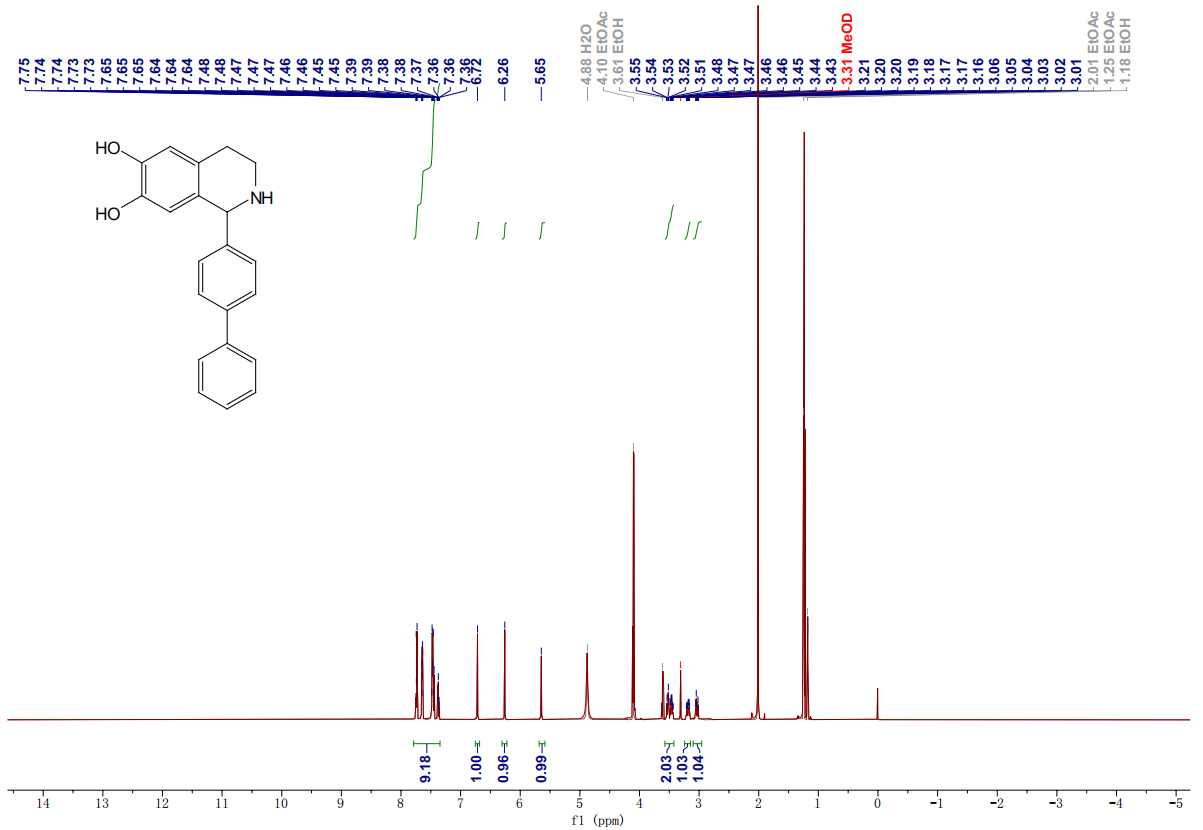


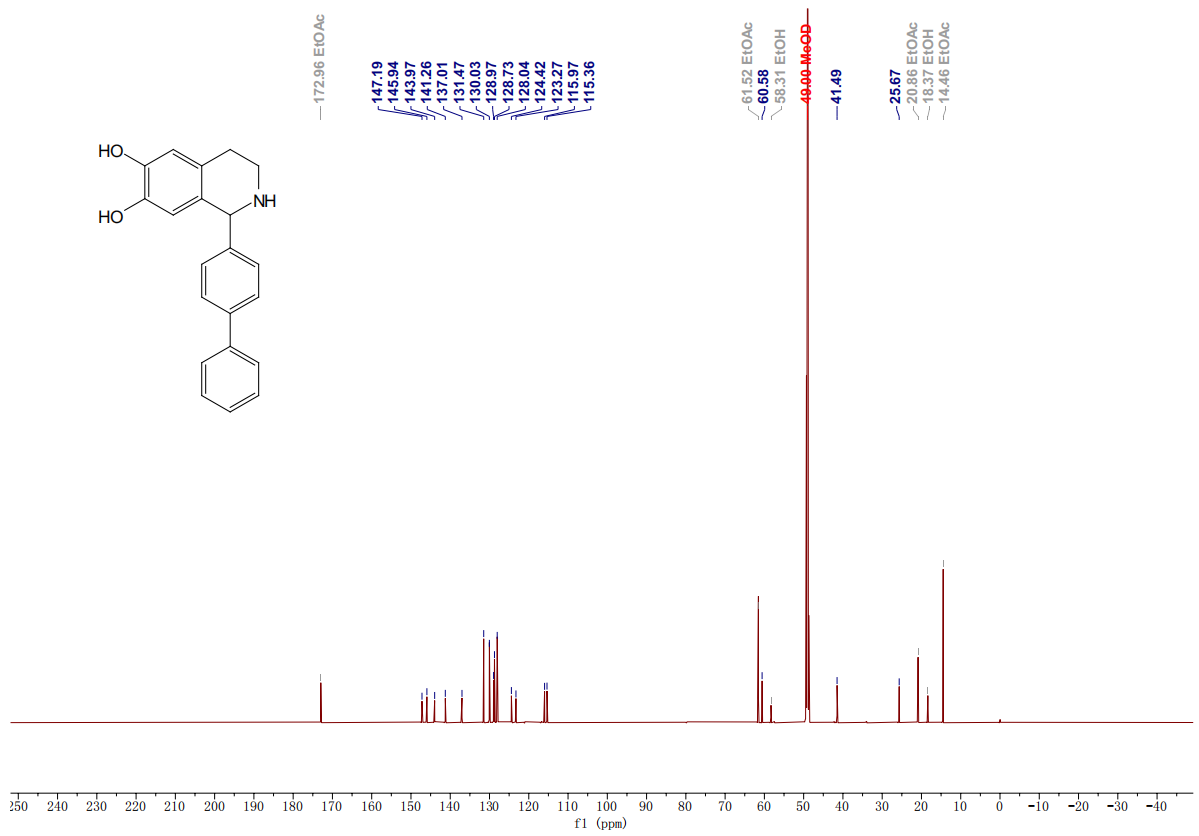


^1^H NMR (600 MHz, CH_3_OD) δ 7.79-7.35 (m, 9H), 6.72 (s, 1H), 6.26 (s, 1H), 5.65 (s, 1H), 3.57-3.42 (m, 2H), 3.19 (m, 1H), 3.03 (m, 1H). ^13^C NMR (151 MHz, CH_3_OD) δ 147.19, 145.94, 143.97, 141.26, 137.01, 131.47, 130.03, 128.97, 128.73, 128.04, 124.42, 123.27, 115.97, 115.36, 60.58, 41.49, 25.67.


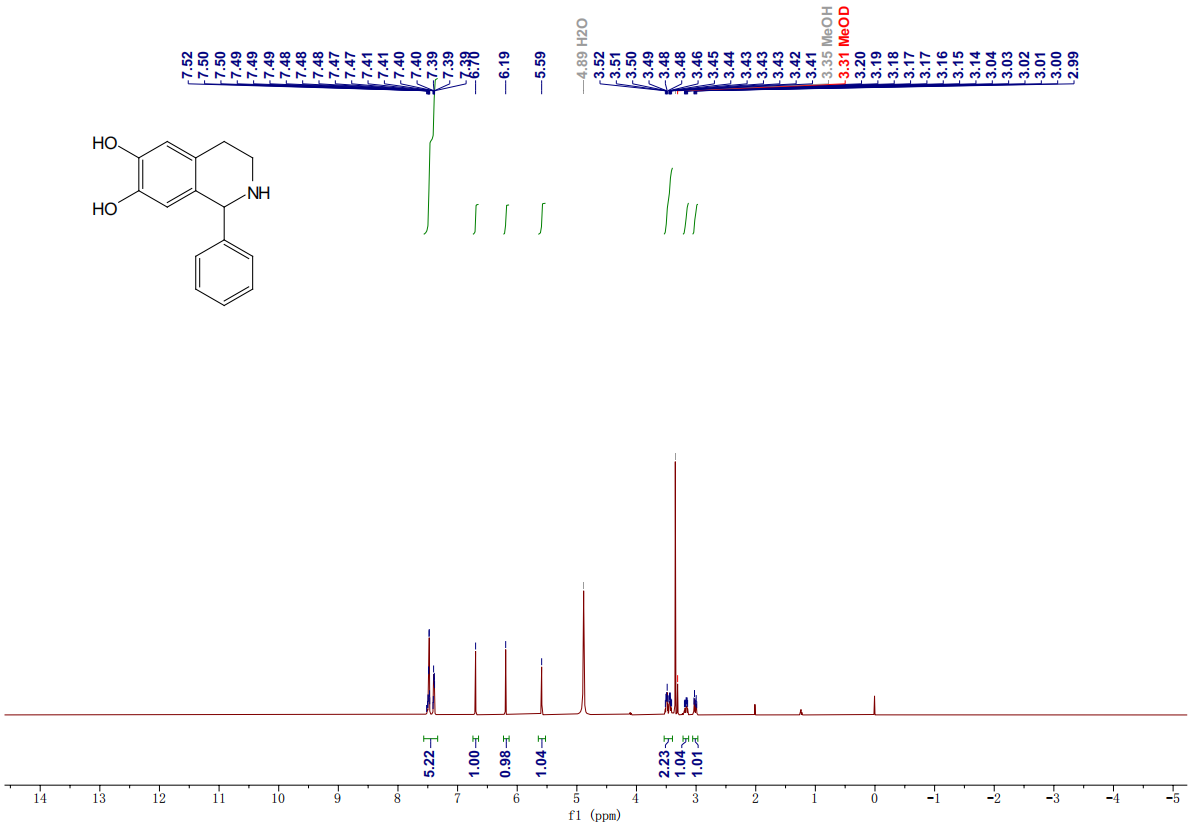


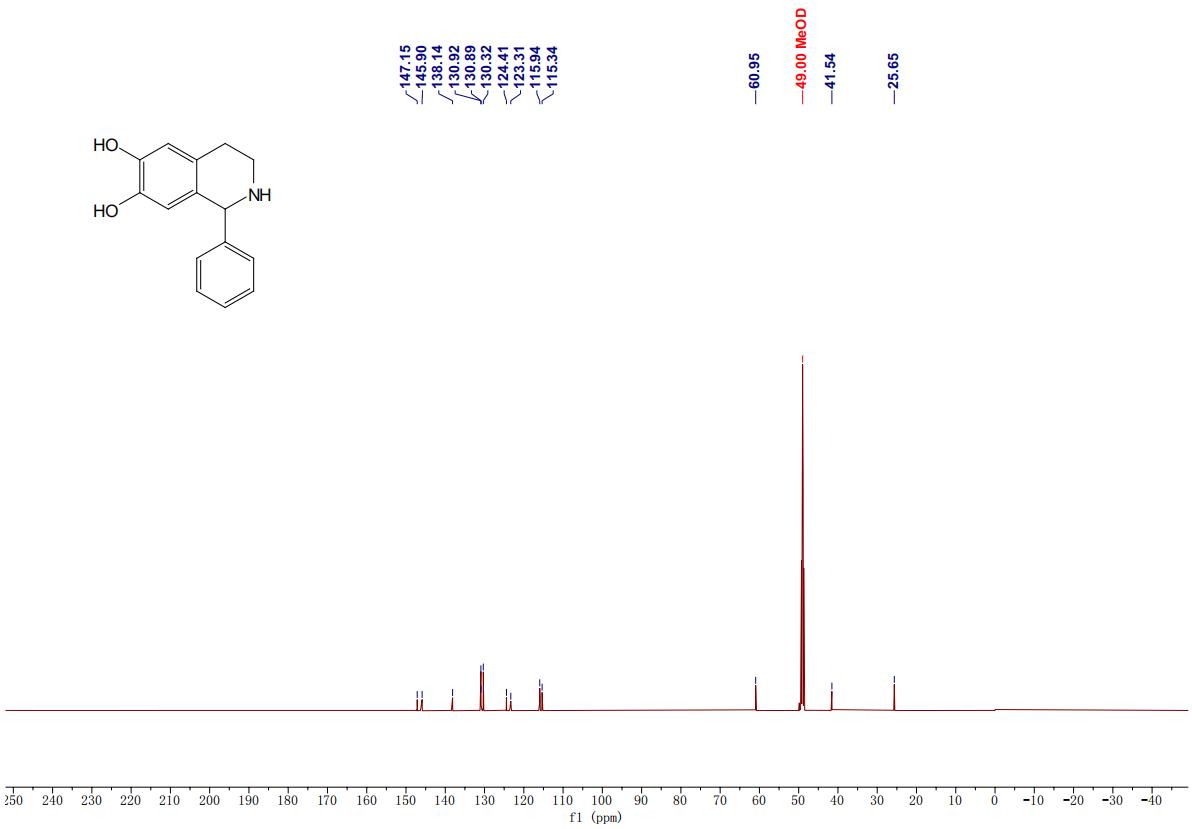


^1^H NMR (600 MHz, CH_3_OD) δ 7.57-7.33 (m, 5H), 6.70 (s, 1H), 6.19 (s, 1H), 5.59 (s, 1H), 3.54-3.40 (m, 2H), 3.17 (m, 1H), 3.01 (m, 1H). ^13^C NMR (151 MHz, CH_3_OD) δ 147.15, 145.90, 138.14, 130.92, 130.89, 130.32, 124.41, 123.31, 115.94, 115.34, 60.95, 41.54, 25.65.

**Figure S8** ^1^H and ^13^C NMR spectra and data of the catalytic product.
